# Rare symbionts may contribute to the resilience of coral–algal assemblages

**DOI:** 10.1038/ismej.2017.151

**Published:** 2017-11-24

**Authors:** Maren Ziegler, Víctor M Eguíluz, Carlos M Duarte, Christian R Voolstra

**Affiliations:** 1 Red Sea Research Center, Division of Biological and Environmental Science and Engineering, 4700 King Abdullah University of Science and Technology (KAUST), Thuwal, Saudi Arabia; 2 Instituto de Física Interdisciplinar y Sistemas Complejos IFISC (CSIC-UIB), Palma de Mallorca, Spain

## Abstract

The association between corals and photosynthetic dinoflagellates (*Symbiodinium* spp.) is the key to the success of reef ecosystems in highly oligotrophic environments, but it is also their Achilles‘ heel due to its vulnerability to local stressors and the effects of climate change. Research during the last two decades has shaped a view that coral host–*Symbiodinium* pairings are diverse, but largely exclusive. Deep sequencing has now revealed the existence of a rare diversity of cryptic *Symbiodinium* assemblages within the coral holobiont, in addition to one or a few abundant algal members. While the contribution of the most abundant resident *Symbiodinium* species to coral physiology is widely recognized, the significance of the rare and low abundant background *Symbiodinium* remains a matter of debate. In this study, we assessed how coral–*Symbiodinium* communities assemble and how rare and abundant components together constitute the *Symbiodinium* community by analyzing 892 coral samples comprising >110 000 unique *Symbiodinium* ITS2 marker gene sequences. Using network modeling, we show that host–*Symbiodinium* communities assemble in non-random ‘clusters‘ of abundant and rare symbionts. *Symbiodinium* community structure follows the same principles as bacterial communities, for which the functional significance of rare members (the ‘rare bacterial biosphere’) has long been recognized. Importantly, the inclusion of rare *Symbiodinium* taxa in robustness analyses revealed a significant contribution to the stability of the host–symbiont community overall. As such, it highlights the potential functions rare symbionts may provide to environmental resilience of the coral holobiont.

## Introduction

Corals and coral reef ecosystems are immediately threatened by global climate change and local anthropogenic impacts (Hoegh-Guldberg *et al.*, 2007). This is because the obligate association of corals with dinoflagellate endosymbionts of the genus *Symbiodinium* represents both the success and the Achilles’ heel of these ecosystems. Fueled by their endosymbiont’s provision of energy in the form of photosynthates, corals provide the structural foundation for the ecologically and economically important reef ecosystems (Roberts *et al.*, 2002). Although reef ecosystems cover only a small percentage of the world’s oceans, almost a third of global marine biodiversity is dependent on their functional integrity (Reaka-Kudla, 1997). But ocean warming and other impacts lead to the breakdown of the coral–*Symbiodinium* symbioses (coral bleaching) and the ongoing decline of coral reef habitats on a global scale (Hoegh-Guldberg *et al.*, 2014; Hughes *et al.*, 2017). Based on this premise, fundamental questions to help forecast the future of coral reefs emerge. For instance, how are *Symbiodinium* communities structured in their respective coral hosts and across reef ecosystems, how will this change under future ocean conditions, and does this affect coral host and ecosystem resilience?

Research on the physiology of *Symbiodinium* is typically focused on the most abundant symbiont(s) within the host (Cooper *et al.*, 2011; Ziegler *et al.*, 2015) or on cultured clonal isolates (Schoenberg and Trench, 1980; Suggett *et al.*, 2015; Parkinson *et al.*, 2016). For instance, the bleaching response and stress tolerance of the coral host are affected by the associated *Symbiodinium* type (Berkelmans and van Oppen, 2006; Abrego *et al.*, 2008), where a type is defined based on ribosomal ITS2 DNA sequences, which, in combination with other genetic markers, is used to distinguish *Symbiodinium* species (LaJeunesse *et al.*, 2012, 2014). However, these host–symbiont associations are not static and may change as a mechanism to increase the size of the ecological niche (Ziegler *et al.*, 2015) or in response to environmental perturbation (Silverstein *et al.*, 2015; Boulotte *et al.*, 2016). The replacement of stress-susceptible with stress-resistant *Symbiodinium* according to the adaptive bleaching hypothesis (Buddemeier and Fautin, 1993) is accomplished by the substitution of dominant symbionts with rare types already present within hosts and within the broader environment (Berkelmans and van Oppen, 2006; Boulotte *et al.*, 2016).

Although most coral colonies are primarily associated with one *Symbiodinium* type at a given time, a large cryptic diversity of low abundant *Symbiodinium* is becoming increasingly apparent through the combined use of next-generation sequencing (NGS) and large-scale sampling of coral–symbiont assemblages (Quigley *et al.*, 2014; Thomas *et al.*, 2014; Hume *et al.*, 2016; Ziegler *et al.*, 2017). These NGS studies have in common that they describe the existence of a rare and diverse *Symbiodinium* community, in addition to an abundant community composed of one or a few symbiont taxa. This observation of few abundant and many rare algal symbionts associated with a coral host parallels the structure of bacterial communities where the so-called rare bacterial biosphere (*sensu* Sogin *et al.*, 2006; Pedrós-Alió, 2012) supposedly represents the predominant sector of bacterial diversity (Skopina *et al.*, 2016). The rare bacterial biosphere has been shown to fulfill essential functions associated with nutrient cycling, the degradation of pollutants, host health, and rare microbes may further also enhance functionality of abundant microbes (reviewed in Jousset *et al.*, 2017). In ocean and freshwater environments, rare members of bacterial and protist communities tend to be more active (as measured by RNA to DNA ratios) and thus contribute overproportionally to ecosystem function (Campbell *et al.*, 2011; Debroas *et al.*, 2015). In the case of corals and their algal endosymbionts, the putative significance of a rare background *Symbiodinium* community remains largely unresolved (Boulotte *et al.*, 2016; Lee *et al.*, 2016). A key question is whether the rare biosphere of microbial symbionts in general, and that of algal endosymbionts in particular, has a functional role in the coral holobiont.

Building onto approaches and insights from other study systems, we further investigated this research question. For instance, the diversity of species and the complexity of their interactions are linked to the stability of ecosystems in a dynamic relationship with environmental drivers (Kondoh, 2003; Ives and Carpenter, 2007). This has been particularly well-studied for plant communities in which phylogenetic and functional species diversity promote ecosystem stability (for example, Cadotte *et al.*, 2012). Mathematical approaches to describe and understand relationships introduce the concepts of (1) ‘networked buffering‘, which relies on community components to perform multiple functional roles and a partial overlap between their functional capabilities (Whitacre and Bender, 2010) and (2) ‘buffered qualitative stability', which predicts robustness of a network (of genes or other biological entities) by its responses to arbitrary (that is, modeled) perturbations (Albergante *et al.*, 2014). Following this, in this study we used network modeling to understand the composition and structure of coral host-associated algal endosymbiont communities. Our aim was to explore the potential role of rare *Symbiodinium* community members in regard to *Symbiodinium* community composition as a whole and in regard to the robustness (that is, stress tolerance) of the coral holobiont community composition under disturbance.

## Materials and methods

### Sample collection and ITS2 sequencing

For the *Symbiodinium* community modeling in this study, we used an NGS data set of the ITS2 rRNA marker gene from 892 coral samples that were previously collected and sequenced from three regions and 22 reef sites around the Arabian Peninsula in the Red Sea, the Sea of Oman, and the Persian/Arabian Gulf (for sample information refer to Supplementary Table S1 in Ziegler *et al.*, 2017). Two sites each were sampled at Subri reef (Persian/Arabian Gulf) and Fahal Island (Sea of Oman), which were previously grouped together, but treated as distinct sampling locations in this study. The *Symbiodinium* ITS2 marker was sequenced using standard methods and quality control was conducted as detailed in Ziegler *et al.* (2017). Briefly, sequence reads were denoised (Quince *et al.*, 2011) and filtered for low-quality scores in mothur v 1.34.1 (Schloss *et al.*, 2009), and then screened for chimeric sequences (Edgar *et al.*, 2011). Because a large proportion of *Symbiodinium* ITS2 sequencing data are comprised of intragenomic ITS2 variants (Arif *et al.*, 2014; Thomas *et al.*, 2014), sequence data were subsampled to 1000 sequences per sample, and clustered into Operational Taxonomic Units (OTUs) at 97% similarity cutoff. This cutoff was shown to capture the taxonomic level typically associated with ‘species’ (or above) (Arif *et al.*, 2014). The final data thus contained information on the abundance of each of 92 *Symbiodinium* OTUs, which ranged in abundance from 1 to 418 068 sequences.

### Evenness of the *Symbiodinium* community: abundance and richness scaling

OTU abundance data were used to create a rank-abundance distribution, where OTUs were ranked by decreasing abundance (that is, the OTU with the highest number of reads received rank 1). Next, we tested for a correlation of the abundance of each OTU with its degree, that is, with the number of samples the respective OTU was found in (the more samples the OTU was found in, the higher the degree). The empirical distribution of OTU abundance indicated that the number of reads per OTU was described by a power-law distribution. These broad distributions require a large range of samples for a proper characterization. To further support this observation, here we developed a theoretical framework that allowed us to relate the richness and abundance of the *Symbiodinium* community with the assumption of a power-law distribution. The scaling of the *Symbiodinium* OTU abundances was best captured by the Yule model (Yule, 1925). In terms of the abundance of OTUs, this model considered two processes: specific mutations, producing new sequences of the same OTU, occurring at a rate *p*, and generic mutations, leading to new OTUs, at a rate *q*. Thus, the governing equations in the continuous limit were:


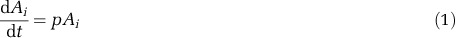



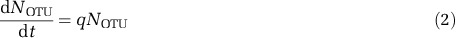


where *A*_*i*_ was the population (abundance) of OTU *i* and *N*_OTU_ was the number of different OTUs. Starting with a single OTU of population 1 at time *t*=0, the solution of these equations led to the following distribution of abundances:


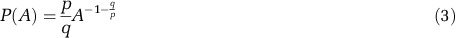


and the population of the most abundant OTU, the initial OTU, was given by 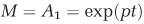, and the total number of different OTUs 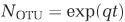. Combining these results we found 
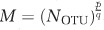
.

The total number of sequences *N* was given by (Simkin and Roychowdhury, 2011)


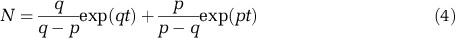


Thus in the limit of large *t*, we obtained for *p*>*q*, *M*~*N* (and thus *N*_OTU_~*N*^*q/p*^), while for *p*<*q*, *N*_OTU_~*N* (and thus *M*~*N*^*p/q*^).

Applying these operations yielded a rank-abundance that scaled as *A*~*r*^-*s*^. From this rank-abundance scaling, the distribution of abundances was given by *P*(*A*)~*A*^−1−1/s^. Comparing with Equation (3), *s*=*p/q*>1 led to the scaling for the most abundant OTU *M*~*N* and the richness *S(=N*_OTU_)~*N*^*1/s*^ (and *S*~*A*^*1/s*^).

### Accounting for ITS2 gene copy number differences

Differences in intragenomic ITS2 gene copy numbers between *Symbiodinium* OTUs may cause a potential bias in our model, as they may lead to over- or underestimation of relative OTU abundance. We addressed this issue by implementing a simulation to account for potential ITS2 gene copy number differences between OTUs. In this approach, OTU counts were first randomly assigned an integer value between 1 and 10 and then the OTU count was randomly multiplied or divided by the assigned value. This corresponds to the reported two orders of magnitude difference in single cell-based intragenomic variability of the ITS2 region in *Symbiodinium* within types (Mieog *et al.*, 2009; Wilkinson *et al.*, 2015) and represents a conservative approach in light of estimated ITS2 copy number ratios of 1:3 to 1:5 between clades (Mieog *et al.*, 2007; unpublished reference in Thornhill *et al.*, 2007). Based on the resulting values for each OTU, we ranked the new relative abundances and re-calculated the OTU community distribution. The simulation was repeated with 1000 iterations to create confidence intervals for an OTU community distribution plot (Supplementary Figure S1). Our approach suggested that the rank of individual low abundant OTUs may change, but that the *Symbiodinium* community structure was not heavily affected by differences in ITS2 copy numbers, which was attributed to the high unevenness of the *Symbiodinium* community.

### Host–*Symbiodinium* bipartite networks and *Symbiodinium* co-occurrence networks

Network modeling was applied to assess composition and structure of coral host-associated algal symbiont communities. To do this, the abundance of each OTU per sample was represented in a bipartite network where each link connected an OTU and a sample with a weight corresponding to the number of sequences. This bipartite network was then transformed into a co-occurrence network where two OTUs were connected if they co-occurred in the same sample and the weight was the number of samples in which they co-occured. The co-occurrence matrix contained 419 links.

Similarly, a bipartite network connecting OTUs and host taxa was constructed by connecting OTUs and the hosts in which they were observed with the total number of sequences. The data contained 46 host taxa. The host co-occurrence network was then constructed by linking pairs of OTUs observed in the same host taxon; the weight reported the number of hosts in which they co-occurred. In this case the host co-occurrence matrix contained 1492 links.

### Identification of co-occurrence clusters

To further elucidate *Symbiodinium* community structure, we tested for statistically significant associations of *Symbiodinium* OTUs that co-occurred with each other in so-called ‘clusters’. We applied Order Statistics Local Optimization Method (OSLOM) (Lancichinetti *et al.*, 2011) as the algorithm to detect topological communities (clusters) from the co-occurrence matrix. OSLOM reported statistical significant clusters for a predefined *P*-value that we set at *P*<0.01. It also reported overlapping OTUs, that is, OTUs could be assigned to more than one community. As the weight informed about the number of co-occurrences we used the ansatz of a weighted network where considering weights as multiple links, which translated into a null model that randomly rewired all these links together with all the links of the network (option –uw).

### Host–*Symbiodinium* community robustness to perturbations

Next, we explored the potential role of rare *Symbiodinium* community members in the robustness (that is, stress tolerance) of the coral host–symbiont community composition under disturbance. We conducted a robustness analysis of the host–*Symbiodinium* network by sequential extinction (that is, removal) of OTUs according to three scenarios: (a) random, (b) generalists, and (c) specialists. In the random scenario, one of the existing OTUs was selected at random for extinction; in the generalist scenario, the OTU with the largest degree (that is, the highest presence over all samples) was selected (in case of >1 OTU complying to this condition, one of them was selected at random); in the specialist scenario, the OTU with the smallest degree was selected (in case of >1 OTU complying to this condition, one of them was selected at random). After selection of an OTU, it was removed from the system together with its links. In cases where samples became devoid of OTUs, the samples also became extinct. After a number of sequential OTU extinctions, the system could split into several connected components. We evaluated the robustness of the host–*Symbiodinium* community against removal of *Symbiodinium* OTUs as a measure of ecosystem resilience against perturbations by quantifying the number of remaining samples connected through the presence of OTUs and the size of largest connected component. All simulations were written in FORTRAN by the authors.

### Adaptive robustness analysis

Recent studies suggest that rare *Symbiodinium* members may replace dominant ones upon environmental change, attesting to a buffering capacity of the *Symbiodinium* community that provides resilience to the host–symbiont system (Boulotte *et al.*, 2016; Hume *et al.*, 2016). To address possible scenarios of emerging rare endosymbionts, we introduced a probability (*p*) that an alternative OTU can take over the prevalence and abundance of a removed OTU. We reduced the set of possible replacement OTUs to those OTUs that originally co-occurred with the removed OTU (in at least one sample). The rationale of this approach was that if the two OTUs co-occurred in at least one sample, there is an increased likelihood that they have close niche spaces allowing the less frequent co-occurring OTU to replace the lost OTU. Proceeding as for the static robustness analysis (above), we then allowed the possibility that each link, indicating the presence of an OTU in a sample, was replaced by another OTU in the adaptive robustness analysis. The replacement was done with probability *p*.

## Results

### *Symbiodinium* community distribution reveals high number of rare species

We explored community patterns of host–*Symbiodinium* assemblages using ITS2 NGS data of *Symbiodinium* from 892 hard corals and other symbiotic anthozoans covering 46 genera across the Arabian Seas (that is, Red Sea, Sea of Oman, Persian/Arabian Gulf) (Ziegler *et al.*, 2017). The resulting data set comprised 92 OTUs, which were clustered from >7 million sequence reads representing >110 000 distinct *Symbiodinium* ITS2 sequences, illustrating the large intragenomic diversity of the ITS2 marker (Ziegler *et al.*, 2017).

**Figure 1:**
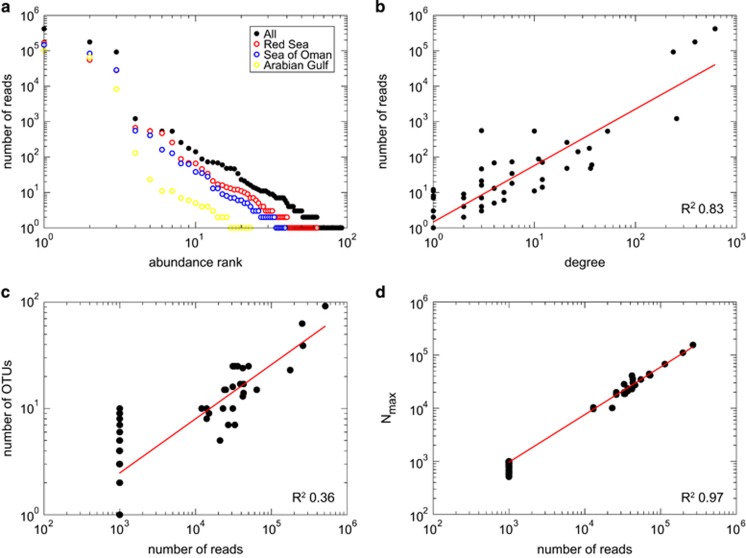
Rank-abundance distribution of *Symbiodinium* taxa (that is, OTUs) in hard corals and other symbiotic anthozoans from the Arabian Seas (**a**) and correlation between presence in coral samples (measured by degree) and the number of reads per OTU (**b**). The power-law relationship indicates that the *Symbiodinium* community is comprised of few abundant and many rare members with a prevalence that scales sub-linearly with abundance, that is, *Symbiodinium* OTUs are not randomly distributed, but concentrated across hosts. (**c**) Richness, *S*, measured as the number of distinct OTUs and (**d**) number of reads of the most abundant OTUs as a function of the total number of reads. The fit shows *S*~*N*^0.5^ and *N*_max_~*N*^0.9^.

The rank-abundance distribution of *Symbiodinium* OTUs in the data set was highly skewed with a few abundant OTUs and a long tail composed of many rare OTUs (OTU abundance range 1-418 068), characterized by a scaling exponent *s*=2.52 (Figure 1a). *Symbiodinium* community composition followed a rank-abundance distribution, best described by a Zipf-like distribution, where the abundance, *A*, scaled with the rank order of the OTUs, *r*, as *A*~*r^–s^*, where *s* was the scaling exponent characterizing the distribution (Clauset *et al.*, 2009; Saichev *et al.*, 2009). In other words, 90% of the less abundant OTUs accounted for less than 1% of the population; in the other extreme, the two most abundant OTUs captured more than 99% of the population (Figure 1a). Thus, the community was composed of few abundant and many rare members (Figure 1a) confirming the *Symbiodinium* diversity to be dominated by a rare symbiont assemblage comparable to the rare biosphere reported for bacteria in microbial communities in the ocean (Pedrós-Alió, 2012) and the human gut (Arumugam *et al.*, 2011). A complementary result on the rarity of the *Symbiodinium* community was obtained by analyzing the scaling of the richness, *S*, and the abundance of the most abundant OTU, *M*, with the total number of sequences (Figure 1c). The agreement of the scaling exponents of the richness and the most abundant OTU with the total number of reads *N* with the rank-abundance distribution based on a power-law distribution supported the hypothesis of a rare coral–symbiont assemblage. Correlation between prevalence in corals, *P* (measured by degree), and abundance (measured by the number of reads) for a symbiont OTU also followed a power-law relationship, *P*~*A^b^* with a scaling exponent, *b*, of 1.59 (±0.08) (s.d.), further providing evidence for a low evenness of OTU-based community structure (Figure 1d). Hence, not only were the majority of the OTUs rare in terms of relative abundance, but they were each also detected in only a small fraction of all coral hosts analyzed, although most host taxa were harboring rare symbiont OTUs (Supplementary Table S1). This resulted in a strong correlation between abundance and prevalence for *Symbiodinium* OTUs (Figure 1b).

### *Symbiodinium* communities form discrete clusters of co-occurrence

**Figure 2:**
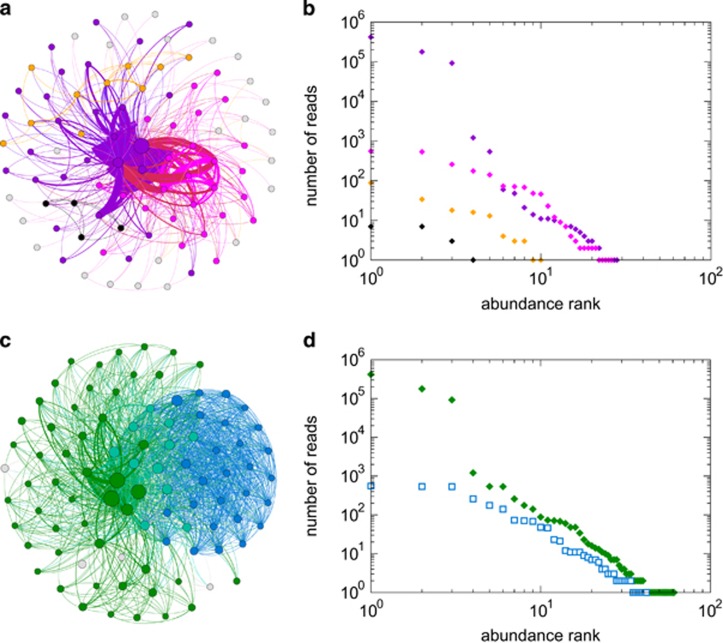
(**a**) Co-occurrence of *Symbiodinium* OTUs in hard corals and other symbiotic anthozoans from the Arabian Seas form four significant clusters (*P*<0.01). Each symbol represents an OTU with respective sizes denoting relative abundance and colors (purple, pink, orange, black) denoting cluster membership; red connections between OTUs represent overlaps between the purple and pink clusters. Lines indicate co-occurrence of two OTUs in a sample, and increasing line thickness indicates a higher number of samples in which the OTUs co-occur. (**b**) The rank-abundance distribution of *Symbiodinium* OTUs in each co-occurrence cluster displays a structured assembly that is comprised of rare and abundant members. (**c**) Two co-occurrence clusters (blue and green) of *Symbiodinium* OTUs were detected when samples were grouped by host taxon, with an overlap of 11 OTUs (turquoise) shared between both clusters. (**d**) The two host taxon-based co-occurrence clusters were of comparable size and followed the power-law scaling behavior of rank abundance. Colors denote separate clusters, colors in (**b**) and (**d**) correspond to clusters in (**a**) and (**c**), respectively, gray=OTUs not part of a cluster (*P* >0.01).

We next examined the patterns of co-occurrence of *Symbiodinium* OTUs at the individual sample (that is, colony) and at the host genus level. The co-occurrence network represented the weighted projection in the OTU set, that is, a link connected two OTUs if they co-occurred in at least one sample (Figure 2a) or host taxon (Figure 2c), and the weight of the link was determined by the number of samples or host genera, respectively, in which they co-occurred. From these co-occurrence networks, we statistically analyzed the *Symbiodinium* community.

Overall, we observed highly structured co-occurrence patterns using the sample-based data set, which resulted in four clusters of OTUs comprising 68 of the 92 OTUs (Figure 2a). The two larger clusters consisted of 28 and 26 OTUs, respectively, and the two smaller clusters consisted of 10 and 4 OTUs. Despite differences in the number of *Symbiodinium* OTUs in the co-occurrence clusters, each of the clusters was equally comprised of rare and abundant OTUs (Figures 2b and d) and conserved the scaling behavior of rank abundance observed for the entire *Symbiodinium* community (Figure 1a). To exclude the possibility that the co-occurrence of abundant and rare OTUs was an artifact caused by the 97% similarity cutoff that may sort intragenomic ITS2 variants into discrete OTUs, we considered the OTUs’ identity based on their most abundant sequence (Supplementary Table S2). The largest co-occurrence cluster consisted of OTUs from five clades (A–D, F), making it unlikely that intragenomic variants were represented in distinct OTUs. Nevertheless, several OTUs had best hits to the same *Symbiodinium* type (for example, C15), indicating that potentially some OTUs may represent intragenomic variants of the same *Symbiodinium* type. The opposite effect may happen if diversity is underestimated with an OTU-based approach, due to the clustering of similar sequences that may *de facto* be representative of distinct *Symbiodinium* types. This might have affected the number and size of the co-occurrence clusters we observed, but potentially has a minor effect on our analysis of *Symbiodinium* community composition because of the large unevenness and robust scaling behavior of the community. When samples were grouped by host taxon, two co-occurrence clusters of *Symbiodinium* OTUs of comparable size were detected (Figure 2c).

### Bipartite host–symbiont networks distinguish specialist and generalist community members

**Figure 3:**
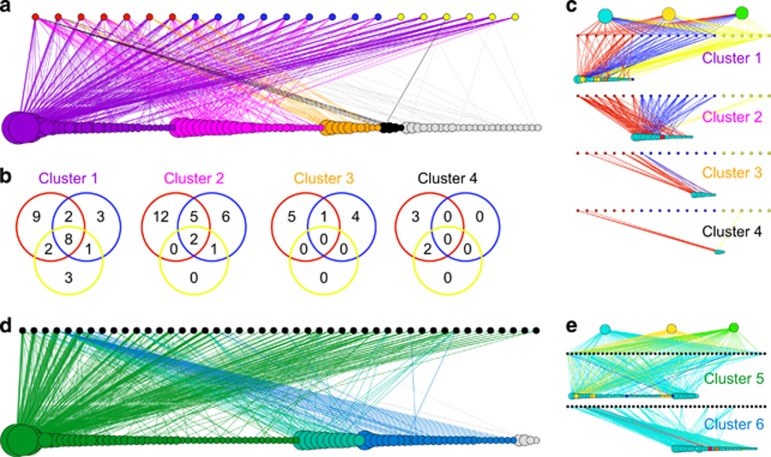
Coral host–*Symbiodinium* bipartite networks over reefs (**a**) and host genera (**d**) from three regions across the Arabian Seas and bipartite networks for significant OTU co-occurrence clusters over reefs (**c**) and hosts (**e**). Top nodes represent reefs over three sampling regions for (**a**, **c**) (red=Red Sea, blue=Gulf of Oman, yellow=Persian/Arabian Gulf), and the number of shared OTUs per region and cluster is illustrated with Venn diagrams (**b**). Top nodes in (**d**, **e**) represent host taxa; all bottom nodes represent *Symbiodinium* OTUs. In (**a**, **d**) OTUs are ordered by cluster (color codes as in Figure 2) and by abundance (indicated by relative size) and in (**c**, **e**) OTUs are ordered by abundance (size) with the three most abundant OTU arranged at the top of the graph for clarity and colored by clade (*Symbiodinium* clade A=green, B=dark orange, C=cyan, D=yellow, F=blue, G=red). Lines represent observed associations. Corals have very few extreme specialists or generalists and *Symbiodinium* have many specialists and a few extreme generalists.

The structure of host–symbiont diversity was examined using bipartite networks, which map the occurrence of reefs or hosts to symbiont OTUs (Figures 3a and d). Our analysis showed that the abundant *Symbiodinium* OTUs seem to represent a resource shared across the Arabian Seas, which were accompanied by many endemic, regional OTUs supporting the argument of the existence of different selective environmental pressures between these regions (Ziegler *et al.*, 2017) (Figure 3b). The largest *Symbiodinium* co-occurrence cluster showed a high degree of mixing between reefs and regions and the three smaller clusters captured more specialized communities that are connected by (that is, share) single OTUs between regions (Figure 3b). *Symbiodinium* phylogenetic diversity increased with cluster size, with the smallest clusters (3 and 4) being comprised only of members of clade C, and clusters 1 and 2 of clade A, B, D, F, and G in addition to clade C, respectively (Figure 3c). The remaining OTUs (*n*=24), not identified as belonging to co-occurrence clusters, were mostly characterized by occurrences in a single reef or host. The host–symbiont networks revealed that coral taxa have very few extreme specialists, in terms of ‘private’ host OTUs, or generalists, in terms of having consistently many OTUs (Figure 3d). *Symbiodinium* in turn has many specialists, that is, OTUs that appeared only in one or a few coral host genera, which were those rare OTUs composing the tail of the rank-abundance plot (Figure 1a), and a few extreme generalists that were found at a high prevalence across different corals and regions and are those with the highest rank abundance in the rank-abundance plot (Figure 3d). Similar to the reef-based clusters, the host taxon-based co-occurrence clusters were comprised of a phylogenetically mixed *Symbiodinium* community, dominated by clade C (Figure 3e).

### Host–*Symbiodinium* community robustness to perturbations

**Figure 4:**
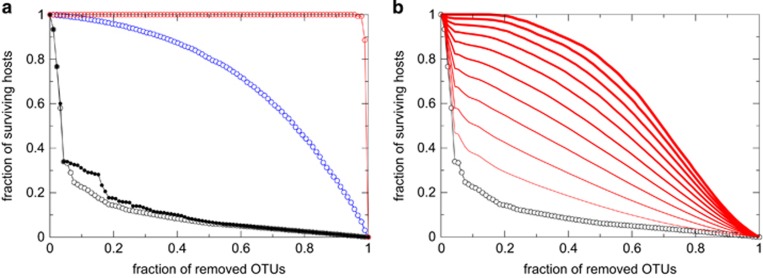
Robustness of the coral–*Symbiodinium* community, measured as the fraction of remaining samples, to removal of *Symbiodinium* OTUs; (**a**) removal strategies depicted: random order (blue), according to their degree (that is, presence in coral samples; white), or according to their abundance (black). Removing components in increasing order of degree (red) shows a more stable scenario compared to removal in random order. When components with the highest degree are removed first (white), the robustness of the system quickly decreases and falls below linear loss of species, indicating that common *Symbiodinium* OTUs contribute overproportionally to maintaining host–symbiont assemblages. (**b**) Allowing for adaptation by introducing the probability that a removed OTU can be replaced by co-occurring OTUs increases the fraction of surviving samples. The probability that an existing OTU replaces the removed OTU increases from 0.1 to 1.0 at intervals of 0.1 from bottom to top (red lines).

We assessed the robustness of the coral system against environmental perturbation by measuring the size of the largest connected component of the bipartite network as OTUs were removed (Figure 4), implying OTUs to differ in their resistance to stress. As OTUs were removed from the bipartite network, the number of hosts represented in the network decreased at rates that were dependent on the removal strategy of the OTUs. The decrease in the number of remaining connected hosts at rates faster than the linear loss expected by random removal occurred when highly connected OTUs, that is, those that associate with many hosts (that is, with high degrees), were removed first. This suggests that host–symbiont assemblages rely disproportionally on common *Symbiodinium* taxa and that these common taxa confer resistance to coral systems against random loss of OTUs from the community (Figure 4a). Our modeling results are based on the assumption of fixed roles of symbiont community members (Lee *et al.*, 2016). However, a host–symbiont community composed of few, common *Symbiodinium* taxa would be rendered highly vulnerable to impacts (for example, disease, environmental extremes) leading to catastrophic ‘mortality’ of the dominant OTUs. Indeed, recent evidence suggests that rare *Symbiodinium* members may replace dominant ones with shifting environmental conditions, therefore providing resilience to host–symbiont communities (Boulotte *et al.*, 2016; Hume *et al.*, 2016). To address possible scenarios of emerging rare members, we introduced a probability (*p*) in an adaptive robustness analysis that an alternative OTU could take over the prevalence and abundance of a removed OTU. Increasing the replacement probability *p* increased the number of surviving hosts, defined as hosts that retain at least one OTU (Figure 4b). For a replacement probability of 10%, a removal of 10% of OTUs led to a doubling in the number of surviving samples (Figure 4b). Consistently, the OTUs responsible for the replacement belonged to the rare proportion of OTUs. The OTUs involved in >50% of the replacements had a prevalence of 1, that is, they only appeared in one sample. The average increase of prevalence for this set was almost 2 (1.96), representing a signature of spreading of rare OTUs upon loss of the dominant OTUs.

## Discussion

The finding of a rare and diverse *Symbiodinium* community within coral hosts parallels the existence of a rare bacterial biosphere (*sensu* Sogin *et al.*, 2006). This cryptic and diverse community structure of *Symbiodinium* within corals was only uncovered recently through the application of NGS approaches. Further, we found this community to be characterized by a rank-abundance pattern following a power-law relationship that matches those reported for planktonic bacterial assemblages (cf. Figure 2 in Pedrós-Alió, 2012). A key question is whether the rare biosphere in general, and that of coral–algal symbionts in particular, have a functional role in the ecosystem (Lee *et al.*, 2016).

Several lines of evidence suggest that the uneven *Symbiodinium* community structure, composed of few abundant and many rare members, may potentially present a so-far overlooked aspect of coral holobiont functioning. First, the prevalent and dominant components of the symbiont community cannot explain the breadth of responses of corals to stress or their resilience to locally restricted environmental perturbations, precisely because the dominant components are shared across wide environmental gradients and across reefs with contrasting status. This further points to the differentiating elements of the assemblage, composed of the rare symbionts, as the components that may underpin such diversity in response. Interactions between members of the bacterial symbiont community can support holobiont functions and traits that cannot be supplied by single taxa in isolation, for example, synergistic effects between pairs of bacterial taxa increase fungal resistance in the freshwater polyp *Hydra* compared to polyps colonized by single bacterial taxa (Fraune *et al.*, 2015). Similarly, the functional roles of *Symbiodinium* assemblages are presumably not carried out by individual OTUs but by assemblages of OTUs, as suggested by the existence of distinct co-occurrence networks involving rare components of the symbiont pool revealed by our analysis. This is in accordance with patterns of co-occurrence of diverse micro- and macroorganisms, which suggests that non-random community assembly supporting ecosystem function and stability may be a general feature of all domains of life (McCann, 2000; Horner-Devine *et al.*, 2007).

Second, rare bacterial community members are shown to fulfill essential functions while at low abundance. They are often more active than abundant members as revealed by rRNA:rDNA ratios and respiration rates (Dimitriu *et al.*, 2010; Campbell *et al.*, 2011). Rare microbes sustain a vast functional gene pool and can indirectly enhance functionality of abundant microbes (reviewed in Jousset *et al.*, 2017). For example, for coral-associated bacterial communities it was suggested that two widely distributed, but low abundant bacterial strains may contribute large proportions of defined metabolic processes within the network of coral host–bacterial functions (Ainsworth *et al.*, 2015). Furthermore, functional diversity of rare species is well documented in assemblages of macroorganisms such as rainforest trees, reef and stream fishes, or birds (Mouillot *et al.*, 2013; Leitão *et al.*, 2016). *Symbiodinium* also represent a highly diverse group that was described on the level of differences between species and between strains within a species (Suggett *et al.*, 2015; Parkinson *et al.*, 2016). Given the large (functional) diversity of *Symbiodinium* and the similarity of *Symbiodinium* community structure to that of bacterial communities (Pedrós-Alió, 2006, 2012), we suggest that similar assumptions can be made regarding the repertoire and contribution of the rare *Symbiodinium* biosphere to community function, and further research should probe the functions provided by the rare *Symbiodinium* biosphere to the coral holobiont while at low abundance.

Further, the network co-occurrence analysis suggests the potential for the rare *Symbiodinium* community to play a role in conferring resistance to the coral host by replacing dominant *Symbiodinium* types lost under environmental stress. The emergence of a regionally cryptic *Symbiodinium* species as prevalent in the heat-selected corals of the Persian/Arabian Gulf (Hume *et al.*, 2016) supports the notion that a phylogenetically diverse and rare algal biosphere may serve as a source of genomic innovation and standing genetic diversity providing the raw material for adaptation to changing environmental conditions (Sogin *et al.*, 2006). As such, an otherwise rare component can become the dominant member and represents a source of adaptation to the coral host–symbiont system (Hume *et al.*, 2016); albeit this reorganization may sometimes come with a trade-off in general ecological performance, such as decreased coral growth rates (Pettay *et al.*, 2015).

The extensive data set used in our study entails samples with mixed communities of *Symbiodinium* that are comprised of combinations of various clade C symbionts, combinations of clade C and clade D symbionts, or clade A with clade C or clade D symbionts in varying abundances. The patterns in these data largely correspond to previously reported switching or shuffling scenarios for clade C to clade D dominance (Jones *et al.*, 2008; Silverstein *et al.*, 2015; Boulotte *et al.*, 2016) or *vice versa* (Jones *et al.*, 2008), between clade C types (Boulotte *et al.*, 2016), and between clade A and C (Hume *et al.*, 2015). In addition, some coral species, such as *Pocillopora verrucosa* and *Porites lutea* (both abundant in this data set) associate with a range of different symbionts throughout reef locations in the Red Sea (Sawall *et al.*, 2015; Ziegler *et al.*, 2015). Thus, they may at least potentially be able to undergo switching or shuffling events under stress scenarios. Despite the large gap of knowledge under which environmental conditions shuffling and switching may occur, which coral species can change their symbionts, or whether this is a selective mechanism, our modeling approach helps exploring the role of rare *Symbiodinium* OTUs in host–*Symbiodinium* community dynamics, thereby providing directions for future research.

The role that *Symbiodinium* background diversity plays in the robustness of the host–symbiont community assembly is a fundamental, but yet unresolved question. Previous models suggested high robustness of highly uneven host–symbiont communities (Fabina *et al.*, 2013), but these conclusions were drawn without the exclusion of intragenomic ITS2 sequence variants that are prone to confound diversity estimates. In our study, we aimed to remove redundancy from intragenomic diversity by applying an OTU-based clustering approach. Consequently, our results suggest a rather low robustness of a static system when dominant *Symbiodinium* components are lost, while rare members remain unchanged and cannot become dominant. We integrated recent studies that suggest that rare *Symbiodinium* community members can become dominant over short time scales following stress events (Boulotte *et al.*, 2016) and during long-term environmental adaptation (Hume *et al.*, 2016) by conducting an adaptive robustness analysis (that is, allowing co-occurring endosymbionts to replace removed members). Comparing the results from both modeling approaches revealed a significant increase in the stability of the host–symbiont community when rare members can become dominant and replace the lost components. Thus, our model highlights a potential role for rare symbionts upon environmental change. It should be noted, however, that although our findings support a potential role of *Symbiodinium* community composition for holobiont functioning at large and for background symbionts as mediating components that buffer the community against stressors in particular, detailed and rigorous experiments should be conducted to confirm this notion.

## Conclusions

Our results reveal a highly structured community assembly in coral host–*Symbiodinium* associations and suggest functional importance of the *Symbiodinium* community as the sum of all of its members, not only of the most dominant members. The host–*Symbiodinium* association is shaped by a few dominant symbionts as well as a rare, diverse community characterized by robust scaling power laws that escaped previous analyses based on reduced sample size and/or sequencing depth. The existence of distinct co-occurrence networks involving rare members of the symbiont pool suggests that the functional role of *Symbiodinium* in the holobiont is not determined by a single individual taxon, but rather by assemblages of symbionts. Intriguingly, the inclusion of rare microbial members in robustness analyses resulted in a significant stability increase of the host–symbiont community and thus further highlights a potential role that rare symbionts may have in corals threatened by environmental change. Given that host–*Symbiodinium* association plays a central role in coral holobiont response to perturbation and resilience, future studies should focus on experimental elucidation of the specific functions contributed to the holobiont by rare *Symbiodinium* members and the synergistic effects arising from their interactions.

### Data accessibility

Sequence data used in this publication can be accessed in the NCBI Sequence Read Archive (http://www.ncbi.nlm.nih.gov/sra) under accession number PRJNA306572.
