# Supplementary material for: Rare symbionts may contribute to the resilience of coral–algal assemblages
Source: ISME J. 2017 Nov 24;12(1):161–72. doi: 10.1038/ismej.2017.151 (PMC5739009; doi:10.1038/ismej.2017.151)
Supplement: Supplementary Information [file ismej2017151x1.docx]

**Supplemental Information**

**Rare symbionts may contribute to the resilience of coral-algal assemblages**

Maren Ziegler^1^, Victor M. Eguiluz^2^, Carlos M. Duarte^1^, Christian R. Voolstra^1,^*

^1^Red Sea Research Center, Division of Biological and Environmental Science and Engineering, 4700 King Abdullah University of Science and Technology (KAUST), 23955-6900 Thuwal, Saudi Arabia

^2^Instituto de Física Interdisciplinar y Sistemas Complejos IFISC (CSIC-UIB), E07122 Palma de Mallorca, Spain

*Corresponding autor/lead contact: [christian.voolstra@kaust.edu.sa](mailto:christian.voolstra@kaust.edu.sa), Tel: +966 12 8082377, Fax: +966 21 8082377

**Supplemental Table S1.** Number of OTUs found in each host taxon and sample of that taxon, and the proportion of the OTUs that were ‘rare/low abundant’ per taxon.

| **Host taxon** | **Region** | **n** | **# of OTUs** | **# OTUs > 10 %** | **# OTUs 1-10 %** | **# OTUs < 1 %** | **mean # OTUs per sample** |
| --- | --- | --- | --- | --- | --- | --- | --- |
| Acanthastrea | Sea of Oman | 3 | 3 | 2 | 0 | 1 | 2.00 |
| Acanthastrea | Red Sea | 1 | 4 | 1 | 1 | 2 | 4.00 |
| Acanthastrea | Persian/Arabian Gulf | 2 | 2 | 1 | 1 | 0 | 2.00 |
| Acropora | Sea of Oman | 22 | 7 | 2 | 0 | 5 | 2.45 |
| Acropora | Red Sea | 47 | 9 | 1 | 1 | 7 | 2.65 |
| Acropora | Persian/Arabian Gulf | 17 | 10 | 2 | 0 | 8 | 2.11 |
| Anemone | Sea of Oman | 2 | 5 | 3 | 0 | 2 | 3.00 |
| Anemone | Persian/Arabian Gulf | 1 | 3 | 1 | 0 | 2 | 3.00 |
| Astreopora | Sea of Oman | 7 | 4 | 1 | 0 | 3 | 2.14 |
| Astreopora | Red Sea | 6 | 6 | 2 | 0 | 4 | 2.67 |
| Cladocora | Red Sea | 1 | 2 | 1 | 0 | 1 | 2.00 |
| Coscinaraea | Red Sea | 2 | 4 | 1 | 0 | 3 | 3.00 |
| Coscinaraea | Persian/Arabian Gulf | 1 | 3 | 2 | 0 | 1 | 3.00 |
| Cyphastrea | Sea of Oman | 8 | 5 | 2 | 0 | 3 | 2.13 |
| Cyphastrea | Red Sea | 2 | 5 | 1 | 0 | 4 | 3.50 |
| Cyphastrea | Persian/Arabian Gulf | 21 | 8 | 1 | 2 | 5 | 2.33 |
| Diploastrea | Red Sea | 5 | 5 | 2 | 0 | 3 | 2.80 |
| Dipsastraea | Sea of Oman | 15 | 7 | 2 | 0 | 5 | 2.60 |
| Dipsastraea | Red Sea | 11 | 7 | 1 | 0 | 6 | 2.36 |
| Dipsastraea | Persian/Arabian Gulf | 17 | 9 | 1 | 1 | 7 | 2.29 |
| Echinophylia | Sea of Oman | 6 | 4 | 2 | 0 | 2 | 2.33 |
| Echinophylia | Red Sea | 1 | 6 | 3 | 0 | 3 | 6.00 |
| Echinophylia | Persian/Arabian Gulf | 3 | 2 | 1 | 0 | 1 | 1.33 |
| Echinopora | Sea of Oman | 8 | 7 | 2 | 0 | 5 | 2.75 |
| Echinopora | Red Sea | 4 | 5 | 2 | 0 | 3 | 3.00 |
| Entacmaea | Persian/Arabian Gulf | 1 | 3 | 1 | 0 | 2 | 3.00 |
| Favites | Sea of Oman | 16 | 6 | 2 | 0 | 4 | 2.75 |
| Favites | Red Sea | 2 | 4 | 1 | 0 | 3 | 2.50 |
| Favites | Persian/Arabian Gulf | 10 | 5 | 2 | 0 | 3 | 1.90 |
| Fungia | Red Sea | 7 | 7 | 1 | 0 | 6 | 2.71 |
| Galaxea | Sea of Oman | 3 | 4 | 2 | 0 | 2 | 2.33 |
| Galaxea | Red Sea | 3 | 4 | 1 | 0 | 3 | 3.00 |
| Gardineroseris | Red Sea | 5 | 5 | 2 | 0 | 3 | 2.40 |
| Goniastrea | Sea of Oman | 3 | 3 | 2 | 0 | 1 | 1.67 |
| Goniastrea | Red Sea | 5 | 8 | 2 | 0 | 6 | 3.40 |
| Goniopora | Sea of Oman | 11 | 4 | 2 | 0 | 2 | 1.82 |
| Goniopora | Persian/Arabian Gulf | 3 | 3 | 1 | 1 | 1 | 2.00 |
| Hydnophora | Sea of Oman | 6 | 6 | 2 | 0 | 4 | 2.33 |
| Leather Coral | Red Sea | 1 | 2 | 1 | 0 | 1 | 2.00 |
| Leptastrea | Sea of Oman | 15 | 8 | 2 | 0 | 6 | 2.67 |
| Leptastrea | Persian/Arabian Gulf | 2 | 5 | 2 | 0 | 3 | 3.00 |
| Leptoria | Sea of Oman | 1 | 3 | 1 | 0 | 2 | 3.00 |
| Leptoria | Red Sea | 1 | 2 | 1 | 0 | 1 | 2.00 |
| Leptoria | Persian/Arabian Gulf | 2 | 2 | 1 | 0 | 1 | 1.50 |
| Lobophyllia | Sea of Oman | 1 | 2 | 1 | 0 | 1 | 2.00 |
| Lobophyllia | Red Sea | 1 | 1 | 1 | 0 | 0 | 1.00 |
| Lobophytum | Red Sea | 3 | 5 | 1 | 1 | 3 | 3.33 |
| Madracis | Persian/Arabian Gulf | 1 | 1 | 1 | 0 | 0 | 1.00 |
| Montastraea | Red Sea | 1 | 1 | 1 | 0 | 0 | 1.00 |
| Montipora | Sea of Oman | 23 | 7 | 3 | 0 | 4 | 2.73 |
| Montipora | Red Sea | 16 | 10 | 3 | 0 | 7 | 2.68 |
| Montipora | Persian/Arabian Gulf | 8 | 5 | 3 | 0 | 2 | 2.00 |
| Mussidae | Persian/Arabian Gulf | 1 | 3 | 1 | 1 | 1 | 3.00 |
| Mycedium | Red Sea | 1 | 3 | 1 | 0 | 2 | 3.00 |
| Nephthea | Red Sea | 1 | 2 | 1 | 1 | 0 | 2.00 |
| Oulophyllia | Persian/Arabian Gulf | 1 | 2 | 1 | 0 | 1 | 2.00 |
| Pavona | Sea of Oman | 12 | 9 | 2 | 1 | 6 | 2.83 |
| Pavona | Red Sea | 10 | 8 | 1 | 1 | 6 | 2.90 |
| Pavona | Persian/Arabian Gulf | 16 | 6 | 1 | 1 | 4 | 1.68 |
| Platygyra | Sea of Oman | 10 | 8 | 2 | 0 | 6 | 2.90 |
| Platygyra | Red Sea | 1 | 4 | 1 | 0 | 3 | 4.00 |
| Platygyra | Persian/Arabian Gulf | 22 | 7 | 2 | 1 | 4 | 2.14 |
| Pocillopora | Sea of Oman | 17 | 7 | 1 | 0 | 6 | 1.88 |
| Pocillopora | Red Sea | 35 | 8 | 1 | 1 | 6 | 2.74 |
| Pocillopora | Persian/Arabian Gulf | 5 | 3 | 1 | 1 | 1 | 2.00 |
| Porites | Sea of Oman | 35 | 19 | 2 | 0 | 17 | 5.00 |
| Porites | Red Sea | 24 | 28 | 1 | 4 | 23 | 5.62 |
| Porites | Persian/Arabian Gulf | 21 | 10 | 1 | 1 | 8 | 2.14 |
| Psammocora | Sea of Oman | 8 | 5 | 1 | 0 | 4 | 2.63 |
| Psammocora | Persian/Arabian Gulf | 12 | 10 | 2 | 0 | 8 | 3.17 |
| Sarcophyton | Sea of Oman | 3 | 7 | 1 | 1 | 5 | 5.00 |
| Sarcophyton | Red Sea | 2 | 10 | 1 | 0 | 9 | 7.00 |
| Seriatopora | Red Sea | 6 | 9 | 2 | 0 | 7 | 3.83 |
| Sinularia | Sea of Oman | 1 | 5 | 1 | 0 | 4 | 5.00 |
| Sinularia | Red Sea | 3 | 8 | 2 | 0 | 6 | 4.67 |
| Stephanocoenia | Red Sea | 1 | 2 | 1 | 0 | 1 | 2.00 |
| Stylocoeniella | Sea of Oman | 1 | 3 | 1 | 0 | 2 | 3.00 |
| Stylophora | Sea of Oman | 16 | 4 | 1 | 1 | 2 | 2.37 |
| Stylophora | Red Sea | 23 | 10 | 2 | 0 | 8 | 3.08 |
| Stylophora | Persian/Arabian Gulf | 3 | 3 | 1 | 1 | 1 | 2.33 |
| Symphyllia | Sea of Oman | 4 | 5 | 1 | 0 | 4 | 3.00 |
| Symphyllia | Red Sea | 1 | 1 | 1 | 0 | 0 | 1.00 |
| Turbinaria | Red Sea | 3 | 5 | 1 | 0 | 4 | 3.00 |
| Turbinaria | Persian/Arabian Gulf | 7 | 6 | 3 | 0 | 3 | 2.14 |
| Xenia | Sea of Oman | 4 | 4 | 1 | 0 | 3 | 2.75 |
| Xenia | Red Sea | 19 | 17 | 2 | 0 | 15 | 3.00 |

**Supplemental Table S2.** Annotation of *Symbiodinium* ITS2 OTUs in four co-occurrence clusters within the sample set of 892 coral samples from sites around the Arabian Peninsula.

| **Cluster #** | **# of OTUs** | **OTU IDs (multiple annotations to same identity)** |
| --- | --- | --- |
| 1 | 28 | A1, A2, B1, C1(x4), C1.5, C3(x2), C3q, C3w, C15(x3), C38, C39(x3), C41(x3), D1, D5, D17, D1a, D3, F4.3 |
| 2 | 26 | C1e, C15(x19), C15a, C15h(x2), C15m, C39, G3 |
| 3 | 10 | C3w, C65(x3), C107(x4), C107b(x2) |
| 4 | 4 | C3, C38, C163a, C163b |


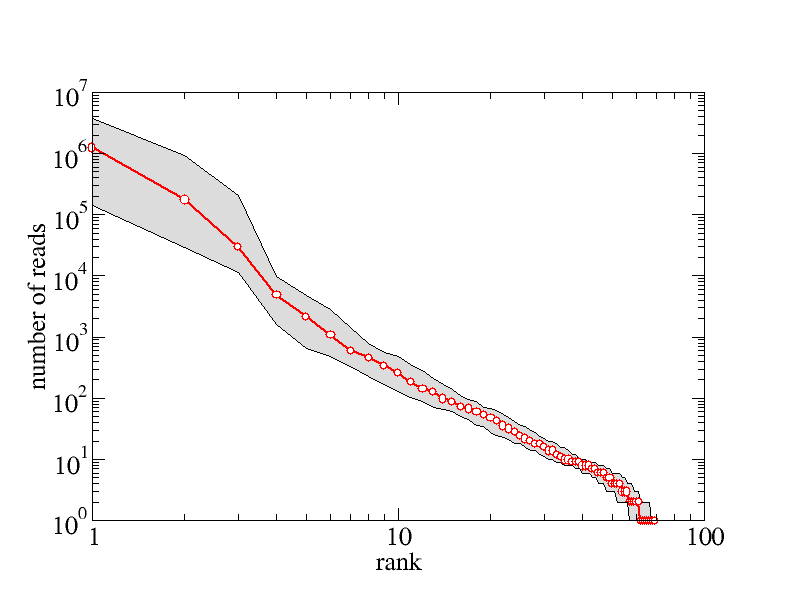


**Supplemental Figure S1.** Simulated rank-abundance distribution of *Symbiodinium* taxa (i.e., OTUs) in hard corals and other symbiotic Anthozoa from the Arabian Seas that accounts for potential differences of ITS2 gene copy numbers between OTUs by two orders of magnitude. Red points and line represent median values and grey shaded area represents the 10 – 90^th^ percentiles after 1 000 simulated iterations.
